# TGF beta1 and related-Smads contribute to pulmonary metastasis of hepatocellular carcinoma in mice model

**DOI:** 10.1186/1756-9966-31-93

**Published:** 2012-11-14

**Authors:** Guo-Cai Li, Qing-Hai Ye, Qiong-Zhu Dong, Ning Ren, Hu-Liang Jia, Lun-Xiu Qin

**Affiliations:** 1 Liver Cancer Institute & Zhongshan Hospital, Institutes of Biomedical Science, Fudan University, Shanghai, China; 2 GaoXin Hospital, Xi’an JiaoTong University, Xi’an, Shanxi Province, China

**Keywords:** Hepatocellular carcinoma, Metastasis, Transforming growth factor beta

## Abstract

**Background:**

Recent studies indicate that Transforming Growth Factor beta (TGF β) correlated with pulmonary metastasis of cancers. However, the correlation between TGF β and pulmonary metastasis of hepatocellular carcinoma (HCC) is till unknown.

**Methods:**

We detected the *in vitro* and *in vivo* expression levels of TGF β1/Smads by Real-time PCR and Western blot in MHCC97-H and MHCC97–L cell lines, which are HCC cell lines and have higher and lower pulmonary metastatic potential respectively.

**Results:**

TGF β1 mRNA level in MHCC97-L tumors were higher than that in MHCC97-H tumors, (2.81±1.61 vs. 1.24±0.96, P=0.002), TGF β1 protein level in MHCC97-L tumors were also higher than that in MHCC97-H tumors (1.37±0.95 vs. 0.32±0.22, P<0.001). In addition, the TGF β1 mRNA level positively correlated with pulmonary metastasis, and the relations between TGF β1 and Smads were also found (R^2^=0.12 and 0.40, respectively).

**Conclusions:**

Our results suggest that TGF β/ Smads promote pulmonary metastasis of HCC.

## Background

Hepatocellular carcinoma (HCC) is one of the most common cancers in the world. The overall five-year survival rate following resection has remained as poor as 35–50% [1-3]. The extremely poor prognosis of HCC is largely the result of a high rate of recurrence after surgery and of metastasis [4,5]. Lung is the most common site for extrahepatic recurrence of HCC. The incidence of pulmonary metastasis after hepatic resection for HCC ranges from 37% to 58% [6]. Therefore, to reduce the pulmonary metastasis could ameliorate the prognosis of HCC.

Transforming growth factor beta (TGF β) is a known regulator of epithelial cell, autonomous tumor initiation, progression and metastasis [7-9]. There are three kinds of molecular in TGF β family, while, TGFβ1 is predominantly and importantly expressed in liver cells [10], whereas two other members, TGFβ2 and TGFβ3, are present in a little amounts and its roles are even ignored in many studies [11]. Signaling of TGF β1 play a role mainly through Smad proteins [12]. Recently, a report indicates that transient exposure of breast cancer cells to TGF β which produced in the primary tumor microenvironment promotes cancer cells to extravagate from blood vessels and entry into the lung by upregulation of the adipokine angiopoietin-like 4 [13].

In HCC, TGF β is a useful serologic marker for diagnosis because it shows higher sensitivity than AFP in earlier stage of cancer [14]. In addition, the role of TGF β1 in HCC metastasis is emphasized. In a study by Giannelli et al. Laminin-5 (Ln-5) and TGF β1 cooperatively induce epithelial mesenchymal transition (EMT) and cancer invasion in HCC [15]. However, although a multitude of studies have presented evidence for TGF β changes in HCC tumors, the direction of the changes is not always consistent. In several studies, TGF β1 levels are demonstrated to be lower [16,17], while, in other studies, the levels are demonstrated to be higher versus healthy individuals [18,19].

In this study, by comparing the different expression of TGF β/Smads in HCC cell lines, we tried to investigate the correlation between TGF β/Smads levels and potential of pulmonary metastasis in HCC.

## Materials and methods

### Cell lines

MHCC97-L and MHCC97-H, were human HCC cell lines, and which have a lower and higher metastatic potential respectively. These cell lines were clonally selected from the same parent cell lines, MHCC97, they have an identical genetic background [20,21]. Both cell lines were cultured in high glucose Dulbecco’s modified Eagle's medium (H-DMEM, Gibco) and supplemented with 10% fetal calf serum (Gibco) at 37°C in a humidified incubator that contained 5% CO_2_.

### Samples

31 samples and observed data were selected randomly from our previous experiment, which were tissues of MHCC97-H models (n=20) and MHCC97-L models (n=11). The models were established as follow: 6×10^6^ MHCC97-H and 6×10^6^ MHCC97-L cells were inoculated subcutaneously into the right side backs of the nude mice (average weight 25g). After tumor formed, the tumor size was estimated according to the formula: volume (mm^3^) = 0.5 a^2^×b, in which “a” is the major diameter of tumor and “b” is the minor diameter perpendicular to the major one [22]. According to our experience, to guarantee enough tumor size and pulmonary metastasis, the MHCC97-L models were feed longer (40days) than MHCC97-H models (35days). In the end of feeding, animals were sacrificed. The tumor and lung tissues were removed and partly cryopreserved in -70°C for real-time PCR analysis, and partly paraffin embedded for immunohistochemstry or H&E (hematoxylin and eosin) staining.

These experiments were approved by the Shanghai Medical Experimental Animal Care Commission, and were in accordance with the Helsinki Declaration of 1975.

### Analysis of pulmonary metastasis

Each lung tissues were sliced for 20 sections with 5μm in thickness, and 50μm interval between two successive sections. After stained with HE, sections were independently observed under microscopic to evaluate pulmonary metastasis by two pathologists.

### RNA extraction and Real-time PCR

Total RNA of MHCC97-H, MHCC97-L cell lines and tumor tissues were extracted by TRIZOL Reagent (Invitrogen corp, USA) according instruction of the product. Real-time RT-PCR analysis was performed to identify the expression level of TGF β1, smad2 and smad7 by using SYBR Green mix(ToYoBo Co, Japan). The primers were designed by software (premier premier 5.0) as follow: TGF β1 (sense 5^′^ GGCGATACCTCAGCAACCG 3^′^; antisense, 5^′^ CTAAGGCGAAAGCCCTCAAT 3^′^), Smad2 (sense, 5^′^ TACTACTCTTTCCCTGT 3^′^; antisense, 5^′^ TTCTTGTCATTTCTACCG 3^′^), Smad7 (sense, 5^′^ CAACCGCAGCAGTTACCC 3^′^; antisense, 5^′^ CGAAAGCCTTGATGGAGA 3^′^), β-actins (sense, 5^′^ -TCGTGCGTGACATTAAGGAG-3^′^; antisense, 5^′^ - ATGCCAGGGTACATGGTAAT-3^′^). Amplification conditions were: 95°C for 9 min, followed by 45 cycles of 95°C for 30s, 57°C for 30s and 72°C for 15s, and followed by an extension at 72°C for 5 min. β-actins was used as a control for the presence of amplifiable cDNA. The mRNA expression level was assessed by 2^-△△Ct^ in brief, the Ct value for target gene was subtracted from the Ct value of β-actins to yield a △Ct value. The average △Ct was calculated for the control group and this value was subtracted from the △Ct of all other samples (including the control group). This resulted in a △△Ct value for all samples which was then used to calculate the fold-induction of mRNA expression of target gene using the formula 2^-△△Ct^, as recommended by the manufacturer (Bio-Rad, Hercules, CA, USA). In this study, we used MHCC97-H model samples as control group. The detection about mRNA expression in MHCC97-H and MHCC97-L cell lines was repeated for 10 times.

### Protein extraction and western blot analysis

1×10^6^ MHCC97-H, MHCC97-L cells and parts of freeze tumor sample (n=12) were lysed in RIPA buffer (50 mM Tris–HCl pH7.5; 150 mM NaCl; 0.5% NaDOC; 1% NP40; 0.1% SDS) plus protease inhibitors. Protein was extracted by micro centrifugation for 30 minutes, Protein concentration was determined using Bradford Reagent. 20ul equal amount of samples and 10ul markers were run onto 10% SDS-PAGE gel and electro-transferred onto PVDF membrane using Mini-Genie blotting system (Bio-Rad). The membranes were incubated with primary antibody, Mouse anti-human TGF β1 antibody (Chemicon, 1:1000 diluted) and Mouse anti-human β-actins antibody (Chemicon, 1:2000 diluted), and HRP-conjugated goat anti-mouse IgG secondary antibody (SIGMA, 1:2000 diluted), The membranes were washed and incubated with 10ml LumiGLO and exposed to film. The blot bands intensity was quantitatively analyzed using FURI Smart View 2000 software (Shanghai). The ratio of TGF β1 to β-actin bands intensity was assessed.

### Cytoimmunochemistry and Immunohistochemistry

2×10^5^ MHCC97-H and MHCC97-L cell were plated and cultured in six-well plate respectively, when reached to 60% confluent, the cells were fixed with 100% methanol, permeabilized with 0.5% Triton X-100, and sequentially incubated with the primary anti- TGF β1 monoclonal antibodies and anti-mouse immunoglobulin (Ig) coupled to Horseradish peroxidase (HRP), then, the cells were stained with DAB (3, 3^′^-diaminobenzidine) and counterstained with hematoxylin. Paraffin-embedded tumor tissues were sliced as 5μm sections in thickness and mounted on glass. Slides were deparaffinated and rehydrated over 10 min through a graded alcohol series to deionized water; 1% Antigen Unmasking Solution (Vector Laboratories) and microwaved were used to enhance antigen retrieval; the slide were incubated with anti-TGF β1 monoclonal antibodies and HRP-conjugated secondary antibody, and then, stained with DAB.

## ELASA

Total protein of all tumor tissues were extracted as described above. TGF β1 protein levels in tumors were determined using the Quantikine TGF β1 Immunoassay (R&D, Minneapolis, MN,USA). The operational approach was performed according to manufacture specification.

### Statistical analysis

Statistical analysis was performed using SPSS 11.5 software (SPSS Inc, USA). The data were analyzed by Students’ *t* test, one-way analysis of variance and covariance analysis. All statistical tests were two-sided; a P value of less than 0.05 was considered statistically significant.

## Results

### The tumor weight and pulmonary metastatic rate

**Figure 1:**
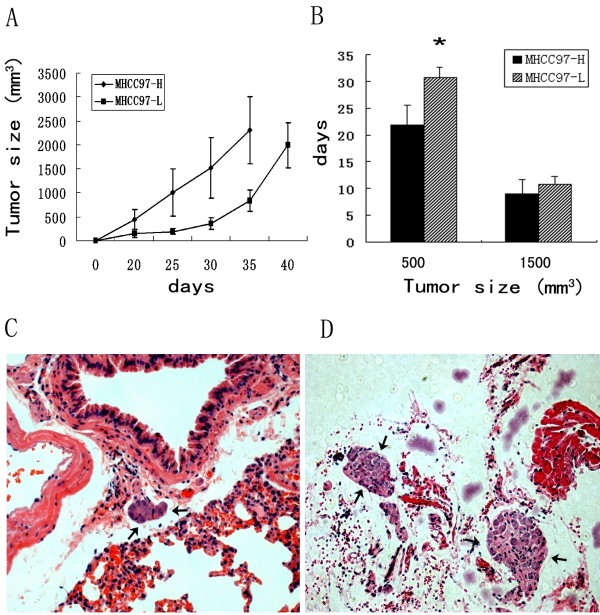
**Comparison of Growth and pulmonary metastsis in mice models. A**) Growth curve of MHCC97-H and MHCC97-L models; **B**) Average days which were spent for getting to tumor size. * denoted P<0.05, Error bar represent the standard errors of the mean. **C,D**) MHCC97-L models (**C**) had smaller pulmonary metastatic loci than MHCC97-H models (**D**). Arrows denote metastatic loci.

The tumors of MHCC9-H model grew fast than that of MHCC97-L, and especially in early stage of tumor formation, MHCC9-H spent shorter time (days) than MHCC97-L getting to the size of 500mm^3^ (21.93±3.67 vs. 30.83±1.94, P<0.001) (Figure 1A), however, the growth speed became similar from the size of 500mm^3^ to 1500 mm^3^ (9.00±2.69 vs.10.83±1.47, P=0.14 ) (Figure 1B). MHCC9-H model had bigger pulmonary metastatic loci than MHCC97-L model (Figure 1C,D). The mean tumor weight (g) in MHCC9-H and MHCC97-L were 1.75±0.75 and 1.26±0.51, and the pulmonary metastatic rate were 55% and 36.36%; and the average number of metastatic cell in lung were 119.25±177.39 and 43.36±47.80 respectively (Table 1).

**Table 1:** The tumor weight and pulmonary metastasis rate in different nude mice models of HCC

**Models**	**No. of cases**	**Tumor weight(g) (Mean±SD)**	**Metastatic rate**	**No. of Metastatic cells (Mean±SD)**
MHCC97-L	11	1.26±0.51	36.36% (4/11)	46.36±47.80
MHCC97-H	20	1.75±0.75	55.00% (11/20)	119.25±177.39

SD=standard deviation.

### The MHCC97-H cells have lower mRNA expression level of TGF β1 and Smads than MHCC97-L in vitro and in vivo

**Figure 2:**
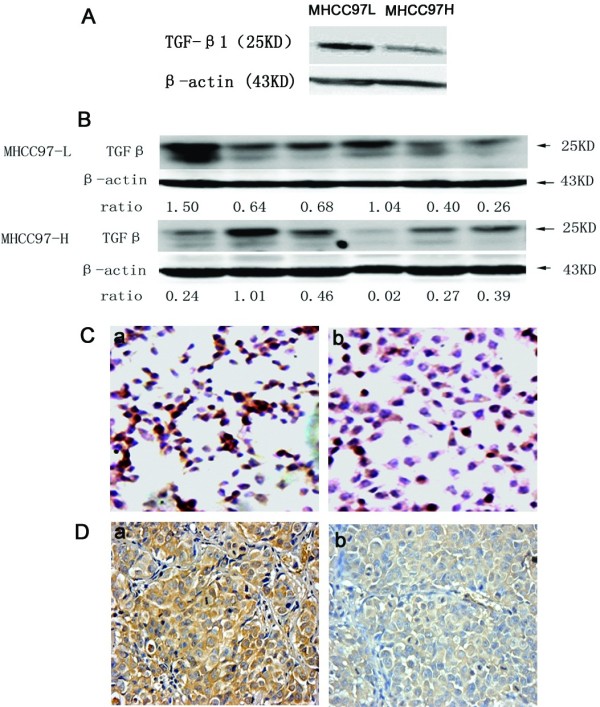
**The TGF β/Smads levels in different cell lines and animal models. A**) The different expression levels of TGF β in MHCC97-H and MHCC97-L by western blot analysis. (**B**). Western blot analysis for tumors. TGF β1 (25KD) and β-actin(43KD) bands of samples from two models. Ratio means: ratio of TGF β1 to β-actin bands intensity. **C**). The different expression levels of TGF β in MHCC97-H and MHCC97-L by cytoimmunochemistry. The brown-yellow color means positive staining, a: MHCC97-L, b: MHCC97-H. (×20 objective field). **D**) The expression of TGF β1 in MHCC97-H and MHCC97-L models by immunohistochemisty staining, the brown-yellow color means positive staining. a: MHCC97-L model, b: MHCC97-H model. (×20 objective field).

As shown in Table 2, mRNA levels of TGF β1 and Smad2 in MHCC97-H cell line were lower than that of MHCC97-L cell line (0.18±0.15 vs. 0.40±0.19, P=0.011; 0.99±0.17 vs. 2.56±0.66, P=0.047), and TGF β1 in MHCC97-H model was also lower than that of MHCC97-L models (1.24±0.96 vs. 2.81±1.61, P=0.002). Compared with MHCC97-L cells, the expression of TGF β1 protein in MHCC97-H was also lower by western blot analysis (Figure 2A), and in mice models, According to quantitative band-intensity analysis of Western blots, the average ratio of TGF β1 to β-actin bands intensity in MHCC97-L models, MHCC97-H models were 0.75±0.45 and 0.57±0.37 (Figure 2B).

**Table 2:** The mRNA expression of TGFβ/Smads in different cell lines and mice models

	**Cell line/ Models**	**MHCC97H or L**	**2-△△Ct (MEAN±SD)**	**95%CI**		**P value**
				**Lower bound**	**Higher bound**	
TGFβ	Cell line	MHCC97H	0.18±0.15	0.07	0.29	
		MHCC97L	0.40±0.19	0.26	0.52	0.011^#^
	Models	MHCC97H	1.24±0.96	0.78	1.69	
		MHCC97L	2.81±1.61	1.73	3.89	0.002*
Smad2	Cell line	MHCC97H	0.99±0.17	0.50	1.56	
		MHCC97L	2.56±0.66	1.38	2.91	0.047^#^
	Models	MHCC97H	1.18±0.73	0.84	1.53	
		MHCC97L	1.52±0.42	1.23	1.80	0.172*
Smad7	Cell line	MHCC97H	12.36±1.62	8.32	16.40	
		MHCC97L	46.98±30.39	−28.52	122.48	0.187^#^
	Models	MHCC97H	1.18±0.62	0.88	1.46	
		MHCC97L	1.48±0.90	0.87	2.08	0.275*

Students’ *t* test was used to assess the statistical significance of differences between two groups. 95%CI: 95% Confidence Interval for Mean, SD=standard deviation, ^#^ compared with MHCC97-H cell line; * compared with MHCC97-H model.

By cytoimmunochemistry (Figure 1Ca, b) and immunohistochemistry method (Figure 2Da, b), we found MHCC97-L cell lines and MHCC97-L models have higher expression level of TGF β1 than MHCC97-H cell lines and MHCC97-H models.

### The TGF β1 protein levels correlated with metastasis

**Figure 3:**
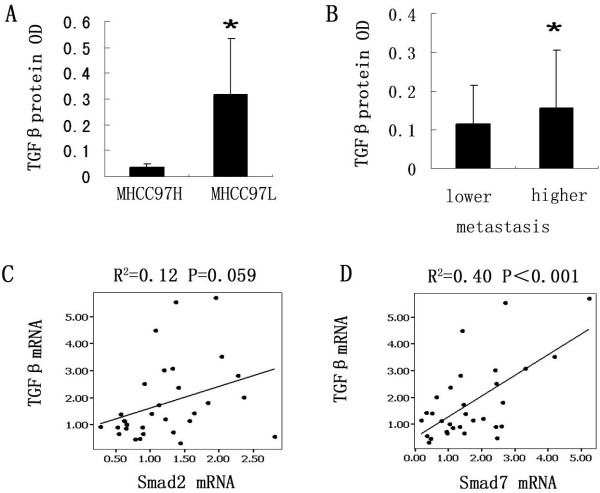
**The expression of TGF β correlated with pulmonary metastasis. A**) MHCC97-L model had a high expression levels than MHCC97-H model by ELASA. * denoted P<0.05. **B**) TGF β1 in metastasis group have higher levels than in non- metastasis group. **C**-**D**) The correlations between TGF β1 mRNA and Smad2, as well as Smad7. Dot denoted the each samples; Lines represent regression line, R: correlation coefficient.

Compared with MHCC97-H models, MHCC97-L models have a higher TGF β1 protein level by ELASA (0.32±0.22 vs. 1.37±0.95, P<0.001) (Figure 3A). And in MHCC97-H and MHCC97-L models, we divided all samples (31cases) into two groups according to metastasis, and we found the TGF β1 protein level in metastasis group was higher than in none metastasis group by covariance analysis (0.16±0.15 vs. 0.12±0.10, P<0.001) (Figure 3B). In addition, in mRNA levels, the relations between TGF β1 and Smad2, Smad7 were also found (R^2^=0.12, P=0.059 and R^2^=0.40, P<0.001, respectively) (Figure 3C,D), but none of them correlated to tumor size.

## Discussion

Although MHCC97-L cell line and MHCC97-H cell line have an identical genetic background, in this study, we observed the expression of TGF β1, Smad2 and Smad7 in MHCC97-L cell lines was higher than that in MHCC97-H cell lines both in vitro and in vivo, in addition, MHCC97-L have a slower growth speed in early stage of tumor formation. Our results were in agreement with other documents, which demonstrate TGF β can induce apoptosis of human hepatoma cell line in vitro [23], and enhance tumor formation by transfection of an antisense TGF-β1 expression vector into cancer cells [24,25]. Our results suggest that the basic level of TGF β in cell line could affect on its growth, and TGF β1/Smads play an inhibitory role in the course of tumorigenensis.

We also found the TGF β1 protein were positively correlated with pulmonary metastasis in the models, and in mRNA levels, TGF β1 correlated with that of Smad2 and Smad7. Our results were consistent with other studies regarding the association between TGF β1/Smads and HCC metastasis [7,15,26], and these results support the veiw that TGF β1/Smads promote pulmonary metastasis of HCC.

The contradict results in this study, inhibitory role in tumorgenesis and promoting role in tumor metastasis, may arise from the dual role of TGF β1 in different stage of cancer development [27]. It has reported during the early stages of tumor formation, TGF β1 acts as a tumor suppressor, inhibiting proliferation and inducing apoptosis of tumor cells. However, during later stages of tumorigenesis, many tumor cells become unresponsive to the growth inhibitory functions of TGF β1, and get more motile, more invasive, and more resistant to apoptosis [13]. In addition, TGF β can stimulate non-invasive HCC cells to acquire invasive phenotypes [28]. Our results support the view that TGF β1/Smads play a dual role in the development of HCC. We also observed MHCC97-L cell lines have a higher TGF β1/Smads levels but a lower metastasis than MHCC97-H cell lines, and both cell lines have an upregulated levels of TGF β1 during the course of metastasis. These results reflected the basic levels of TGF β1 were not the only factor for metastasis, and highlight that the role of TGF β1/Smads should be decided in an active course.

The result that TGF β correlate with pulmonary metastasis in our study will give a new insight to investigate the metastatic mechanism of HCC. The cells in the tumor tissue communicate through the secretion of growth factors, chemokines, and cytokines during tumor progression, and TGF β is unique in its ability to both promote and inhibit tumorigenesis, depending on the cell type it is acting on [29]. Moreover, TGFβ1 could affect various molecular expression, such as P160^ROCK^[30], Integrin [31] and Matrix Metalloproteinases [32],and all of these molecules relate to HCC invasion.

## Conclusions

Collectively, our results suggest that TGF β1 play an important role in the process of tumor growth and pulmonary metastasis of HCC, and the role were time-dependent and based on cell type itself. Strategies to modulate expression levels of TGF β1 could provide a better approach for the treatment of pulmonary metastasis in HCC.

## Abbreviations

HCC: Hepatocellular carcinoma; PCR: Polymerase chain reaction; DMEM: Dulbecoo's modified Eagle's medium; PBS: Phosphate buffered saline; AFP: Alpha fetal protein.

## Competing interests

The authors declare that they have no competing interests.

## Authors’ contributions

GCL wrote the first draft of the manuscript, performed cell culture and contributed to the experimental design; QHY analyzed data and contributed to writing and editing of the manuscript; DQZ and NR analyzed data; LXQ designed experiments and wrote and revised the manuscript; HLJ performed western blot and wrote and edited the manuscript. All authors read and approved the final manuscript.

## Authors’ informations

This work was supported in part by China National Natural Science Foundation for distinguished Young Scholars (30325041), the China National '863' R & D High-tech Key Project.
